# Adipose-Derived Stem Cells From Patients With Ulcerative Colitis Exhibit Impaired Immunosuppressive Function

**DOI:** 10.3389/fcell.2022.822772

**Published:** 2022-02-18

**Authors:** Xiaoyun Wu, Yongxu Mu, Jingyi Yao, Fuhong Lin, Daocheng Wu, Zhijie Ma

**Affiliations:** ^1^ Key Laboratory of Biomedical Information Engineering of the Ministry of Education, School of Life Science and Technology, Xi’an Jiaotong University, Xi’an, China; ^2^ Department of Technology, Research Center for Hua-Da Precision Medicine of Inner Mongolia Autonomous Region, Hohhot, China; ^3^ Department of Interventional, The First Affiliated Hospital of Baotou Medical College, Inner Mongolia University of Science and Technology, Baotou, China; ^4^ Experimental Center, Beijing Clinical Research Institute, Capital Medical University Affiliated Beijing Friendship Hospital, Beijing, China; ^5^ Department of Neurology, Affiliated Hospital of Chifeng College, Chifeng, China; ^6^ Department of Pharmacy, Capital Medical University Affiliated Beijing Friendship Hospital, Beijing, China

**Keywords:** autologous stem cell transplantation, adipose stem cells, immunosuppression, autoimmune disease, mesenchymal stem cells

## Abstract

Adipose-derived stem cells (ADSCs) are able to modulate the immune response and are used for treating ulcerative colitis (UC). However, it is possible that ADSCs from patients with inflammatory or autoimmune disorders may show defective immunosuppression. We investigated the use of ADSCs from UC patients for autologous cell treatment, specifically, ADSCs from healthy donors (H-ADSCs) and UC patients (P-ADSCs) in terms of various functions, including differentiation, proliferation, secretion, and immunosuppression. The efficacy of P-ADSCs for treating UC was examined in mouse models of acute or chronic colitis. Both H-ADSCs and P-ADSCs were similar in cell morphology, size, adipogenic differentiation capabilities, and cell surface markers. We found that P-ADSCs had lower proliferative capacity, cloning ability, and osteogenic and chondrogenic differentiation potential than H-ADSCs. P-ADSCs exhibited a diminished capacity to inhibit peripheral blood mononuclear cell proliferation, suppress CD25 and CD69 marker expression, decrease the production of inflammation-associated cytokines interferon-γ and tumor necrosis factor-α, and reduce their cytotoxic effect on A549 cells. When primed with inflammatory cytokines, P-ADSCs secreted lower levels of prostaglandin E_2_, indoleamine 2, 3-dioxygenase, and tumor necrosis factor-α–induced protein 6, which mediated their reduced immunopotency. Moreover, P-ADSCs exhibited weaker therapeutic effects than H-ADSCs, determined by disease activity, histology, myeloperoxidase activity, and body weight. These findings indicate that the immunosuppressive properties of ASCs are affected by donor metabolic characteristics. This study shows, for the first time, the presence of defective ADSC immunosuppression in UC, indicating that autologous transplantation of ADSCs may be inappropriate for patients with UC.

## Introduction

Inflammatory bowel disease (IBD) primarily comprises Crohn’s disease (CD) and ulcerative colitis (UC). Both occur throughout the world, may result in death, and appear to be increasing in Asian countries(Su et al., 2018). Recent studies indicate that both innate and adaptive immunities participate in disease pathogenesis. Various chemokines and inflammatory cytokines are also associated with IBD(Verstockt et al., 2018).

Mesenchymal stromal cells (MSCs) have immunomodulatory capabilities, suggesting their potential in the treatment of UC(Ciccocioppo and Corazza, 2018; Hosseini-Asl et al., 2020). MSCs are found in bone marrow (BM), but the collection of BM may result in pain and risk of infection in patients. Additionally, with an increase in age, the number, proliferation, and differentiation ability of MSCs in BM gradually decline(Myneni et al., 2018). Recently, there have been several reports that autologous BM-derived MSCs (BMMSCs) derived from patients with nonmalignant hematological diseases (Kuci et al., 2020), diabetes (Jin et al., 2010; Kim et al., 2015; Zhu et al., 2019), rheumatoid arthritis (Sun et al., 2015), systemic lupus erythematosus (Nie et al., 2010; Guo et al., 2015), multiple sclerosis (Redondo et al., 2018), and other immune disease states may show different immunomodulatory characteristics in pediatric patients. However, some studies have shown that BMMSCs derived from patients with acute myeloid leukemia (de la Guardia et al., 2018), CD (Bernardo et al., 2009), severe idiopathic nephrotic syndrome in children (Starc et al., 2018), type 1 diabetes mellitus (Yaochite et al., 2016), and chronic pancreatitis (Wang et al., 2019) are morphologically similar, with comparable proliferative, differentiation, and immunosuppressive capabilities to MSCs derived from healthy donors. These different results suggest that autoimmune diseases affect MSCs immunomodulation, possibly the result of differences in immune cell and cytokine activities.

Adipose tissue (AT) is a major organ of the human body. Compared with the BM, AT can be obtained from several regions of the body and fat harvesting causes less pain and trauma to the donor. Moreover, our research group as well as others have observed that adipose-derived stem cells (ADSCs) and BMMSCs have different biological characteristics. ADSCs have stronger proliferation ability and immunomodulatory function compared with BMMSCs (Bortolotti et al., 2015; Li et al., 2015), and their safe and effective use in treating various diseases has been shown(Tsuchiya et al., 2017; Bateman et al., 2018). These findings also bring hope to patients with severe UC or refractory UC patients who have failed conventional treatment(De Francesco et al., 2016; Adak et al., 2017; Ciccocioppo et al., 2019). As an important endocrine organ, AT participates in the body’s energy regulation, inflammation, and immune response. AT may also actively contribute to the pathological inflammation seen in IBD pathology(Goncalves et al., 2015; Karaskova et al., 2021). Several studies have reported that the inflammatory disorders caused by obesity and type 2 diabetes (Serena et al., 2016), osteoarthritis (Skalska and Kontny, 2016), atherosclerosis (Kizilay Mancini et al., 2017), and rheumatic diseases (Kuca-Warnawin et al., 2019) alter the immune properties of ADSCs, possibly contributing to their reduced immunomodulatory characteristics. Recent studies have reported increased ADSC numbers in IBD patients(Mizushima et al., 2018). ADSCs from Crohn’s disease patients were found to protect the colonic epithelium in mice(Hoffman et al., 2018). However, the immune dysfunction in UC and Crohn’s disease differs. Therefore, whether the immunomodulatory function of ADSCs is affected by the disease state of UC has not yet been reported.

Recent studies have reported that intestinal epithelia from UC patients differ in terms of epigenetics and transcriptional activity (Howell et al., 2018), with high RNA and protein expression of TLR-2 and TLR-4 in myofibroblasts, crypt cells, and stem cells(Brown et al., 2014). In this study, we examined the ADSCs from UC patients (P-ADSCs) and healthy individuals (H-ADSCs) in terms of phenotype, colony formation, multilineage differentiation, proliferation, secretion, immunosuppression, and therapeutic efficacy in a dextran sulfate sodium (DSS)-induced mouse model of acute or chronic colitis with the aim of determining their suitability for autologous cell therapy.

## Materials and Methods

### Donor Information and Sample Collection

The cases for this study were inpatient individuals with UC chosen between January 2013 and March 2014 who underwent autologous ADSC transplantation at the Beijing Friendship Hospital affiliated to the Capital Medical University. Among them, there were three males and four females with an average age of 47.14 ± 8.28 years and body mass index (BMI) of 21.51 ± 2.14 kg/m^2^. The disease course was 21–42 months with an average of (33.29 ± 7.36) months. Patients with UC were classified based on the Truelove-Witts standards and included one mild cases, three moderate cases, and three severe cases. The inclusion criteria of this study were as follows: ①Patients diagnosed with UC according to the “Consensus on the Diagnosis and Treatment of Inflammatory Bowel disease” promulgated by the Chinese Medical Association in 2012 as the diagnostic criteria and diagnosed with UC using colonoscopy and histopathological examination and ②complete clinical material. The exclusion criteria were as follows: ①Patients with other infections; ②patients with other intestinal diseases; ③patients with immune system diseases and malignant tumors. Patient characteristics are presented in Supplementary Table S1. Additionally, healthy donors with matched gender (three males and four females), age (44.37 ± 5.28 years, *p* > .05) and BMI (22.67 ± 2.25 kg/m^2^, *p* > .05) during the same period were used as controls. Biopsy samples of subcutaneous abdominal fat (250–300 mg) were collected from healthy donors or patients with UC using an 18 G needle. All participants in the study provided written informed consent. The study was approved by the ethics committee of Beijing Friendship Hospital affiliated to the Capital Medical University.

### Isolation and Culture of ADSCs

H-ADSCs and P-ADSCs were enzymatically isolated, as previously described (Li et al., 2015). Briefly, AT samples were washed with phosphate-buffered saline (PBS) in a 50 ml Falcon tube and digested with 50 ml of 0.2% collagenase type IV (Sigma, United States) at 37°C for 30 min. The cells were centrifuged (300 x g, room temperature) to obtain stromal vascular cells, which were then cultured at a density of 2×10^5^ cells/mL in 5% human platelet lysate (hPL, Stemery, China)-supplemented medium. Media were replenished after 2 days, and the non-adherent cells were discarded. Thereafter, media were replaced twice weekly. Both types of cells were passaged using trypsin-EDTA when 80–90% confluent and seeded at 3,000 cells/cm^2^.

### Cell Sizes and Doubling Times

The viability of P-ADSCs or H-ADSCs at passage five was determined by trypan blue staining. The numbers and sizes of the cells were assessed with a Cellometer Auto T4 (Nexcelom) with the provided software. The population doubling time (PDT) was calculated using the formula: “PDT = t × [lg2/(lgNt-lgNo)], where t = culture time, Nt = final cell number, and No = initial cell number”.

### Colony-forming Unit-Fibroblast (CFU-F) Assay

At passage 5, P-ADSCs or H-ADSCs were re-plated in 35-mm dishes at 100 cells per dish in 5% hPL. Fourteen days later, the cells were washed in PBS, fixed in cold 100% methanol for 15 min, and stained with 0.5% crystal violet (Beyotime, Jiangsu, China) for 10 min. CFU-Fs (colonies of over 50 cells) were counted under optical microscopy.

### Flow Cytometry

P-ADSCs or H-ADSCs at passage five were harvested, and single-cell suspensions were incubated with antibodies against CD14, CD19, CD90, CD105, CD34, CD45, CD73, and HLA-DR. Detailed information about antibodies is provided in Supplementary Table S2. Cells were analyzed on a flow cytometer (FACSCalibur), with CellQuest Pro 3.7 (Becton Dickinson, San Jose, CA, United States).

### Multilineage Differentiation and Staining Assay

Passage-5 P-ADSCs or H-ADSCs were assayed for chondrogenesis, osteogenesis, and adipogenesis using kits, and stained according to a previous protocol(Ellingsgaard et al., 2011). Reagents were obtained from Cyagen (Guangzhou, China).

### Real-Time Polymerase Chain Reaction (PCR)

RT-PCR was conducted using Stratagene Mx3000P (Agilent Technologies) with QPCR MxPro v4.10d software as previously described (Wu et al., 2020a). The primers are shown in Supplementary Table S3. Relative gene expression was analyzed using the 2^-∆∆Ct^ method with *β*-actin as the housekeeping gene.

### Inflammatory Priming Assay

Passage-5 P-ADSCs or H-ADSCs were seeded at 5×10^4^ cells/well in 6-well plates for 12 h, and then treated with 10 ng/ml interferon-γ (IFN-γ) and 10 ng/ml tumor necrosis factor-*α* (TNF-α) separately or combined for 48 h. The contents of prostaglandin (PG)E_2_, interleukin (IL)-6, and TNF-α–induced protein 6 (TSG-6) in the culture media were analyzed using enzyme linked immunosorbent assay (ELISA) kits (R and D Systems, United States), according to supplied instructions, and indoleamine 2,3-dioxygenase (IDO) activity (kynurenine concentration) was measured as previously described (Wu et al., 2020b).

### Lymphocyte Inhibition Assay

Peripheral blood mononuclear cells (PBMCs) were collected from two different healthy donors. P-ADSCs or H-ADSCs at passage five were treated with 10 μg/ml mitomycin C (Sigma Aldrich,United States) for 2 h, co-cultured with PBMCs in a 1:5, 1:10, or 1:20 ratio with 1 μg/ml anti-CD3/anti-CD28 antibodies (Beijing T&L Biological Technology Co.,Ltd. China) and 200 U/mL recombinant IL-2 (Beijing T&L Biological Technology Co.,Ltd. China) for 72 h. Proliferation was measured using a cell counting kit-8 assay (Beyotime, Jiangsu, China), and the absorbance at 450 nm was measured in a microplate reader. Inhibition of proliferation was directly proportional to the PBMCs without ADSCs. The proliferation rate of PBMCs was calculated using the formula: “OD _(experimental group)_/OD _(PBMCs without stimulus)_ × 100%”.

### Cell Cycle Detection

After 72 h of treatment, PBMCs were harvested and subjected to flow cytometry. Cells were washed, fixed in 70% ethanol overnight at 4°C, and collected by centrifugation. After addition of rnase A (15 μL of 400 μg/ml) for 20 min at 37°C, 3 μg/ml of propidium iodide (30 min at 4°C, Life Technologies, United States) and DNA contents measured by flow cytometry using ModFIT software.

### Detection of Lymphocyte Activation Molecules

The cell density of PBMCs co-cultured for 72 h from different groups was adjusted to 1×10^6^/ml, and 10 µL of CD25 and CD69 antibodies were added, respectively. Detailed information about antibodies is provided in Supplementary Table 2. Mouse IgG-FITC and IgG-PE were included in the isotype control tube and incubated at 4°C for 30 min. The cells were washed, collected by centrifugation, resuspended in 500 µL of PBS and analyzed using flow cytometry.

### Detection of Inflammation-Related Proteins

The cell supernatants from different groups cultured for 72 h were clarified by centrifugation and IFN-γ, TNF-α, IL-10, and transforming growth factor-β1 (TGF-β1) levels were determined using the respective ELISA kits according to the manufacturers’ instructions (R&D Systems, United States).

### Detection of the Killing Ability of PBMCs

Three types of cells, ADSCs, PBMCs, and GFP-A549, were co-cultured in Transwells. By measuring the proliferation ability of A549 cells in each group, the effect of each group of ADSCs on the killing function of PBMCs of GFP-A549 cells was evaluated. The specific procedure was as follows: PBMCs (lower layer) and A549 cells (upper layer) were seeded in a 10:1 cell ratio of PBMC:A549, and P-ADSCs or H-ADSCs were seeded in the lower layer. In addition, 1 μg/ml anti-CD3 monoclonal antibody, 1 μg/ml anti-CD28 monoclonal antibody, and 200 U/mL IL-2 were added as stimuli to the culture system and incubated for 72 h. Then, 100 μL of the upper layer was added to a 96-well plate, after which CCK-8 was added. The culture plate was incubated for 2 h and the OD at 450 nm was determined. Lastly, the killing rate of each group of PBMCs to A549 was calculated using the formula: (OD _(blank group)_-OD _(experimental group/control group)_)/OD _(blank group)_ × 100%.

### Colitis Induction and Treatment

Acute colitis was induced in 6–8-week-old male C57BL/6 mice using 5% DSS (MP Biochemicals, China) added to the drinking water for seven consecutive days unless the animals had to be humanely sacrificed in the event of severe bleeding(Fuenzalida et al., 2016). Intraperitoneal injection of 1×10^6^ P-ADSCs or H-ADSCs in 100 μL PBS was administered to each mouse and their weights were measured each day. Mice that had not received DSS were used as controls (naive). Each experiment was conducted using ADSCs from different donors, with seven animals per group.

For the chronic colitis model, mice received 3% DSS in their drinking water for 5 days in three cycles (5 days DSS treatments on days 0–5, 10–15, and 20–25, followed by a 5-day recovery period after each treatment). On days 7, 17, and 27, mice were intraperitoneally injected with P-ADSCs or H-ADSCs (1×10^6^ cells/mouse).

The disease activity index (DAI) was determined as a combination of weight loss, consistency of the stool, and degree of bleeding (Supplementary Table S4). Colons were removed at euthanasia, separated from cecum to anus, and measured. Myeloperoxidase (MPO, Zeye Biotechnology, China) was measured in colons as previously described(Liu et al., 2019).

### Histological Evaluation

Colon samples were fixed (4% paraformaldehyde) dehydrated, and paraffin-embedded. Sections (5 µm) were stained with hematoxylin and eosin (H and E) and evaluated under a light microscope. The sections were scored by two blinded experienced pathologists in terms of damage to epithelia, crypt loss, and inflammatory cell infiltration (Supplementary Table S5).(Ma et al., 2019).

### Statistical Analysis

Data represent means ± standard deviation (SD). Differences between two groups were analyzed by the Wilcoxon-Mann-Whitney nonparametric test, and Kruskal–Wallis one-way analysis of variance (ANOVA) were used for assessing multiple-group differences. *p* < .05 was considered statistically significant as indicated in each case (*indicates *p* < .05, **indicates *p* < .01 and ***indicates *p* < .001). SPSS 17.0 was used for analyses.

## Results

### Characteristics of P-ADSCs and H-ADSCs

**FIGURE 1:**
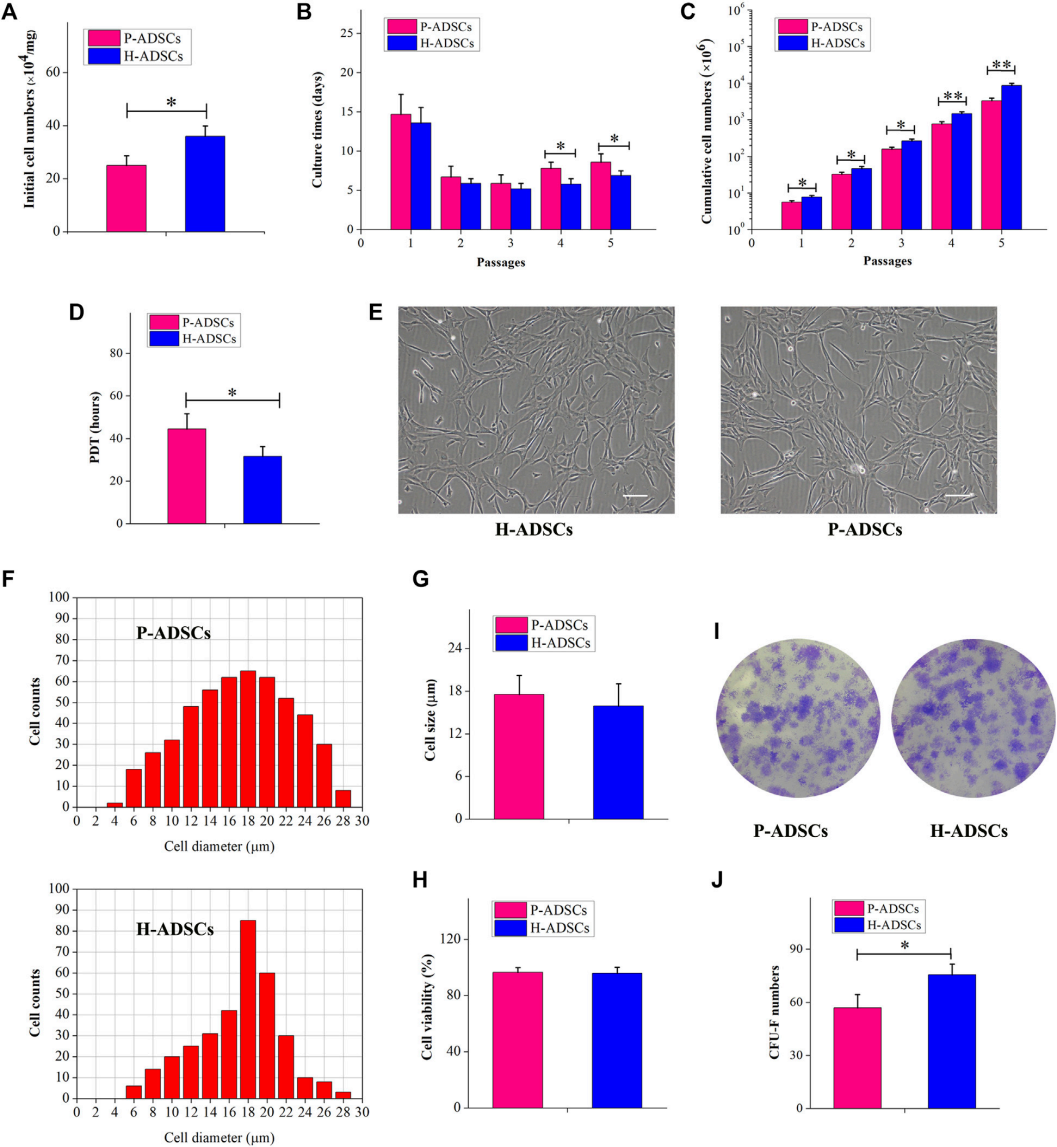
Morphology and self-renewal characteristics of P-ADSCs and H-ADSCs **(A)**: Initial cell number per mg of AT cultivated in 5% hPL. *n* = 7; *p < .05 determined by the Wilcoxon-Mann-Whitney nonparametric test **(B)**: Culture days after a series of passages. *n* = 7; *p < .05 determined by the Wilcoxon-Mann-Whitney nonparametric test **(C)**: Cumulative cell numbers as a function of passage. *n* = 7; *p < .05 and **p < .01 determined by the Wilcoxon—Mann-Whitney nonparametric test. **(D)**: Population doubling time of P-ADSCs or H-ADSCs. *n* = 7; *p < .05 determined by the Wilcoxon-Mann—Whitney nonparametric test **(E)**: Morphological characteristics of P-ADSCs or H-ADSCs cultivated in 5% hPL at passage 5. Representative images are shown. Scale bar = 100 μm. **(F)**: Cell diameter histogram of P-ADSCs or H-ADSCs at passage 5. The cell diameter of monodispersed cells was also determined by image analysis, using the Cellometer Auto T4. Representative images are shown. **(G)**: Cell sizes of P-ADSCs or H-ADSCs. **(H)**: Cell viability of P-ADSCs or H-ADSCs cultivated in 5% hPL at passage 5. **(I)**: Crystal violet-stained after 14 days in culture. Representative images are shown (×4) **(J)**: CFU-F numbers of P-ADSCs or H-ADSCs cultivated in 5% hPL at passage 5 (*p < .05 and **p < .01; *n* = 7).

Fewer P-ADSCs were obtained per mg of AT than H-ADSCs (*p* < .01, Figure 1A). Moreover, the initial incubation periods for the two types of cells did not differ significantly, at about 12–17 days (*p* > .05, Figure 1B). To evaluate the proliferative capacity of P-ADSCs and H-ADSCs, ADSCs were further propagated for five passages. H-ADSCs required fewer culture days at the fourth and fifth passages (both *p* < 0.05, Figure 1B), but exhibited higher overall numbers at each subculture (passages 1–3: *p* < .05; passages 4–5: both *p* < .01; Figure 1C) compared with P-ADSCs. Moreover, H-ADSCs exhibited lower average PDT than P-ADSCs (*p* < .05, Figure 1D). P-ADSCs and H-ADSCs expanded in hPL at passage five exhibited slender and bright fibroblast-like morphology typical of MSCs (Figure 1E). Cell size was calculated as the mean cell diameter, and viability was measured by trypan blue exclusion. Both types of cells were of similar size (*p* > .05, Figures 1F,G) and viability (*p* > .05, Figure 1H). The CFU-F assay showed that, while both P-ADSCs and H-ADSCs supported clonal expansion at passage 5 (Figure 1I), there were significantly fewer CFU-Fs among P-ADSCs (*p* < .05, Figure 1J).

### Immunophenotype of P-ADSCs and H-ADSCs

**FIGURE 2:**
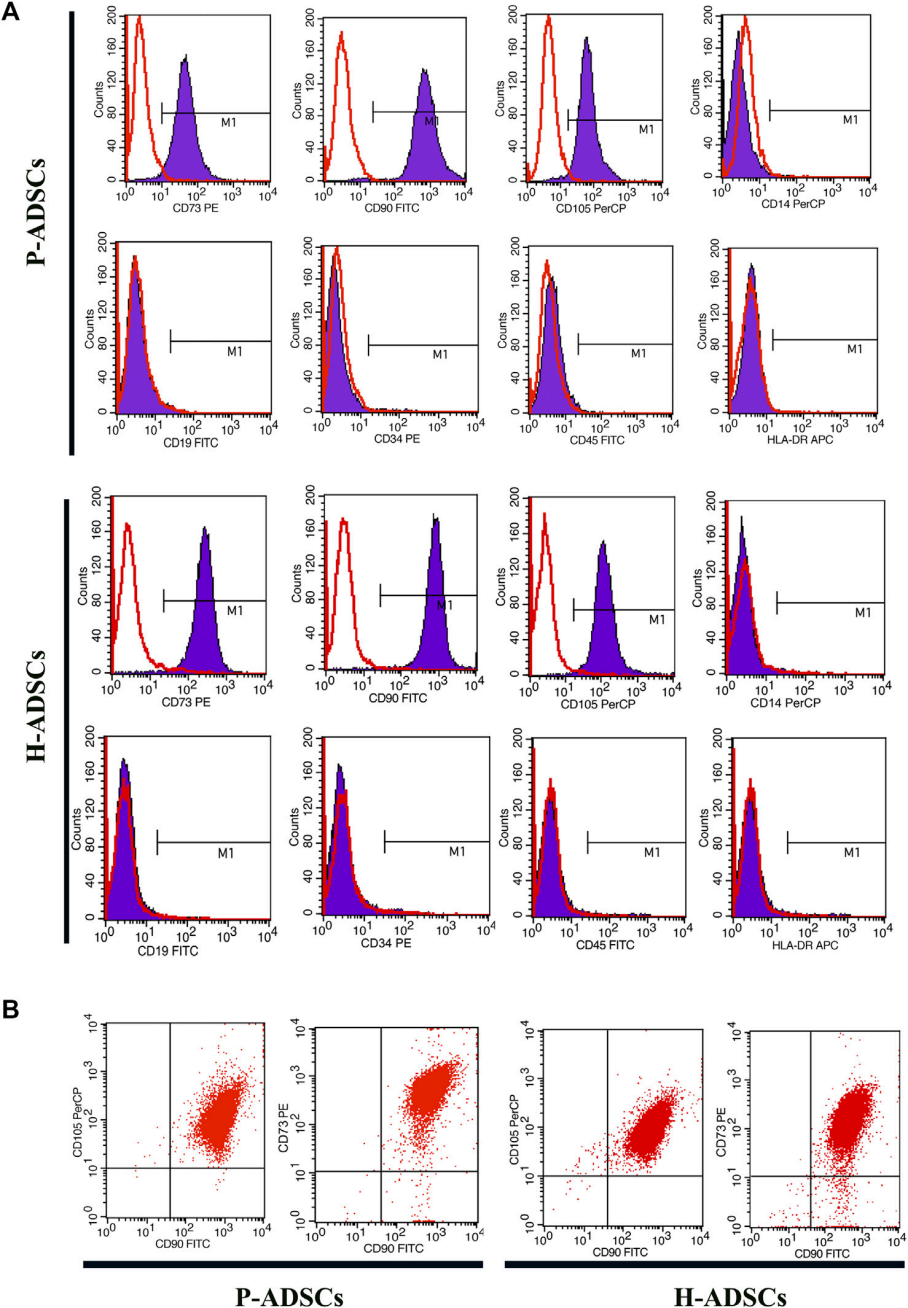
Comparison of phenotypic expansion in P-ADSCs and H-ADSCs **(A)**: Histogram plots showing the surface positive marker panel (CD73, CD90, and CD105) and negative marker panel (CD14, CD19, CD34, CD45, and HLA-DR). Representative images are shown **(B)**: Dot plot showing CD73/CD90 and CD90/CD105 staining. Representative images are shown.

Flow cytometry revealed P-ADSCs or H-ADSCs in hPL at passage five to have a minimal expression (<2%) of CD14, CD19, CD34, CD45, and HLA-DR, but high expression (>95%) of CD73, CD90, and CD105 surface molecules (Supplementary Figure S1; Figure 2A), with a high positive percentage for double stained population CD73/CD90 and CD90/CD105 (>95%, Figure 2B). Moreover, the two cell types did not differ significantly (all *p* > .05, Table 1). In addition, two cell types also highly expressed triple positive CD73/CD90/CD105 (>95%, data not shown).

**TABLE 1:** Surface marker expression levels of P-ADSCs and H-ADSCs at passage five in hPL.

Surface marker	Expression level (%)		p Value
	P-ADSCs	H-ADMSCs	
CD14	1.52 ± 0.29	1.37 ± 0.24	P> .05
CD19	1.08 ± 0.31	0.86 ± 0.44	P> .05
CD34	1.01 ± 0.37	1.18 ± 0.51	P> .05
CD45	1.62 ± 0.47	1.80 ± 1.02	P> .05
HLA-DR	1.33 ± 0.85	1.28 ± 0.92	P> .05
CD73	97.62 ± 2.32	98.61 ± 1.85	P> .05
CD90	98.67 ± 1.44	97.95 ± 1.65	P> .05
CD105	98.29 ± 1.93	97.78 ± 1.69	P> .05
CD73/CD90	97.66 ± 2.29	96.36 ± 2.23	P> .05
CD90/CD105	98.18 ± 1.65	97.28 ± 1.49	P> .05

Data are expressed as mean ± SD.

### Differentiation Potentials of P-ADSCs and H-ADSCs

**FIGURE 3:**
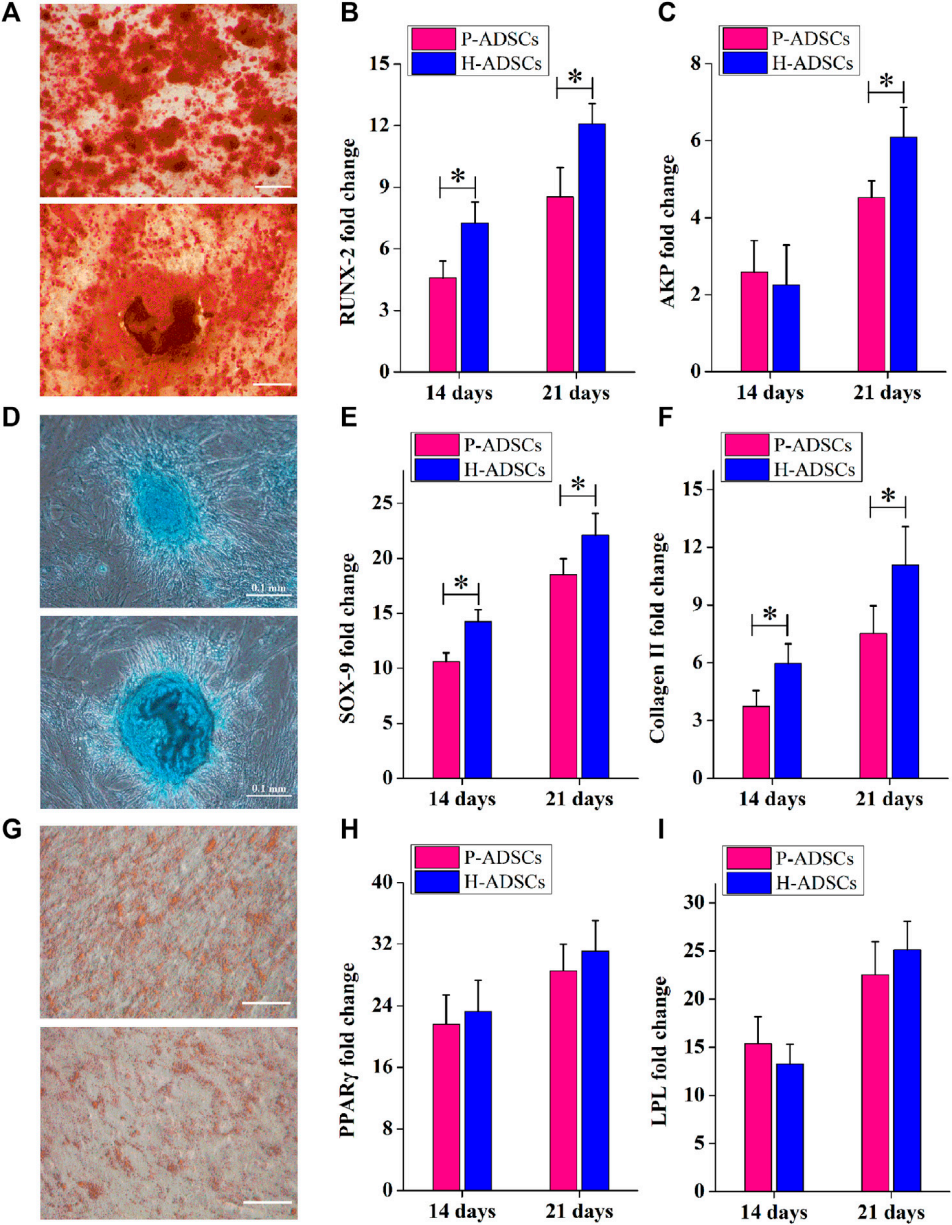
Comparison of the differentiation potential of P-ADSCs and H-ADSCs **(A)**: Osteogenic differentiation of P-ADSCs or H-ADSCs cultivated in 5% hPL at passage 5, after different inductions and stained with alizarin red. Representative images are shown. Scale bars = 100 μm. **(B,C)**: Levels of osteogenic-related genes determined by RT-PCR. n = 7; *p < .05 determined by the Wilcoxon-Mann-Whitney nonparametric test. **(D)**: Chondrogenic differentiation of P-ADSCs or H-ADSCs cultivated in 5% hPL at passage 5, after different inductions and stained with Alcian blue. Representative images are shown. Scale bars = 100 μm **(E,F)**: Levels of chondrogenic-related genes determined by RT-PCR. *n* = 7; *p < .05 determined by the Wilcoxon-Mann-Whitney nonparametric test **(G)**: Adipogenic differentiation of P-ADSCs or H-ADSCs cultivated in 5% hPL at passage 5 after different inductions and stained with oil red O. Representative images are shown. Scale bars = 100 μm. **(H–I)**: Levels of adipogenic-related genes determined by RT-PCR (*n* = 7).

Alizarin red staining showed that P-ADSCs or H-ADSCs expanded in hPL at passage five differentiated into osteogenic cells (Figure 3A). mRNA levels of runt-related transcription factor 2 (RUNX2) at days 14 and 21 after differentiation (both *p* < .05, Figure 3B) and alkaline phosphatase (AKP) at day 21 (*p* < .05, Figure 3C) revealed that P-ADSCs had significantly reduced potential for osteogenic differentiation than H-ADSCs.

The ability of P-ADSCs or H-ADSCs to differentiate into chondrogenic lineages was investigated using Alcian blue staining (Figure 3D). The relative expression of sex-determining region Y-box 9 (SOX-9) and collagen Ⅱ at days 14 and 21 after differentiation, indicated that P-ADSCs at passage five had a significantly lower chondrogenic potential than H-ADSCs (all *p* < .05, Figures 3E,F).

Similarly, Oil Red O staining indicated that P-ADSCs or H-ADSCs at passage five could differentiate into adipogenic lineages (Figure 3G). Moreover, there were no significant differences in peroxisome proliferator-activated receptor gamma (PPARγ, both *p* > .05, Figure 3H) and lipoprotein lipase (LPL, both *p* > .05, Figure 3I) expression between the two types of cells at days 14 and 21 after differentiation, indicating that P-ADSCs exhibited a similar adipogenic differentiation potential as H-ADSCs.

### Immunosuppressive Capacity of P-ADSCs and H-ADSCs

**FIGURE 4:**
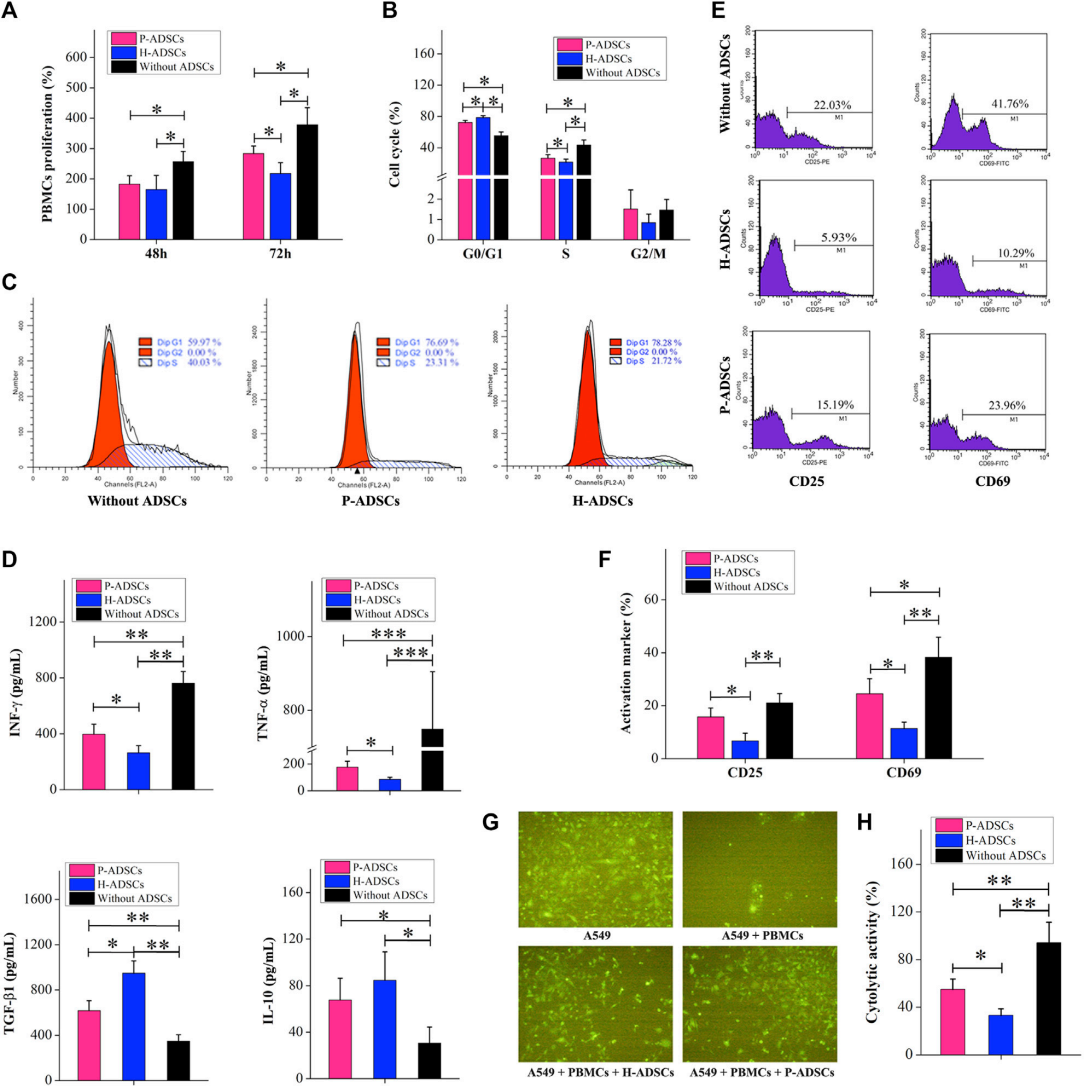
Comparison of the immunosuppressive capacities of P-ADSCs and H-ADSCs **(A)**: Proliferation rate of PBMCs co-cultivated with P-ADSCs or H-ADSCs. *n* = 7; *p < .05 determined by the Kruskal–Wallis one-way analysis **(B)**: Cell cycle status of PBMCs co-cultivated with P-ADSCs or H-ADSCs. *n* = 7; *p < .05 determined by the Kruskal–Wallis one-way analysis **(C)**: Representative images of the cell cycle of PBMCs co-cultivated with P-ADSCs or H-ADSCs **(D)**: Expression levels of the inflammatory factors IFN-γ, TNF-α, TGF-βl, and IL-10 of PBMCs co-cultivated with P-ADSCs or H-ADSCs. n = 7; *p < .05, **p < .01, and ***p < .001 determined by the Kruskal–Wallis one-way analysis **(E)**: Representative histograms of activated markers CD25 and CD69 of PBMCs co-cultivated with P-ADSCs or H-ADSCs **(F)**: Expression level of CD25 and CD69 of PBMCs co-cultivated with P-ADSCs or H-ADSCs. *n* = 7; *p < .05 and **p < .01 determined by the Kruskal–Wallis one-way analysis **(G)**: Representative immunofluorescence images of A549 co-cultivated with PBMCs/P-ADSCs or H-ADSCs. (100×) **(H)**: Cytotoxicity of PBMCs on A549 co-cultivated with P-ADSCs or H-ADSCs. *n* = 7; *p < .05 and **p < 0.01 determined by the Kruskal–Wallis one-way analysis.

Compared with H-ADSCs, co-culturing of P-ADSCs and PBMCs for 48 h affected the ability of MSCs to inhibit PBMC proliferation, but the difference was non-significant (*p* > .05, Figure 4A). When the co-cultivation time was increased to 72 h, compared with H-ADSCs, the effect of P-ADSCs on the proliferation ability of PBMSCs was significantly different (*p* < .05, Figure 4A). We further tested and found that after 72 h of co-cultivation, the proportion of PBMCs in the G0/G1 phase in the P-ADSCs group was lower than seen in the H-ADSC group (*p* < .05, Figures 4B,C). However, the percentage of S-phase PBMCs among P-ADSCs was higher (*p* < .05, Figures 4B,C), while no significant differences were apparent in the G2/M phase (*p* > .05, Figures 4B,C). IFN-γ and TNF-α expression in P-ADSCs was higher than in H-ADSCs (both *p* < .05, Figure 4D), whereas TGF-βl expression was lower than in H-ADSCs (*p* < .05, Figure 4D). However, the levels of IL-10 were essentially the same in both groups (*p* > .05, Figure 4D). CD25 and CD69 levels in PBMCs in the P-ADSC group were lower than in H-ADSCs (Supplementary Figure S2; both *p* < .05, Figures 4E,F). In addition, compared with P-ADSCs, H-ADSCs reduced the cytotoxic effect of PBMCs on A549 (*p* < .05, Figures 4G,H).

### Immunosuppressive Mediators of P-ADSCs and H-ADSCs

**FIGURE 5:**
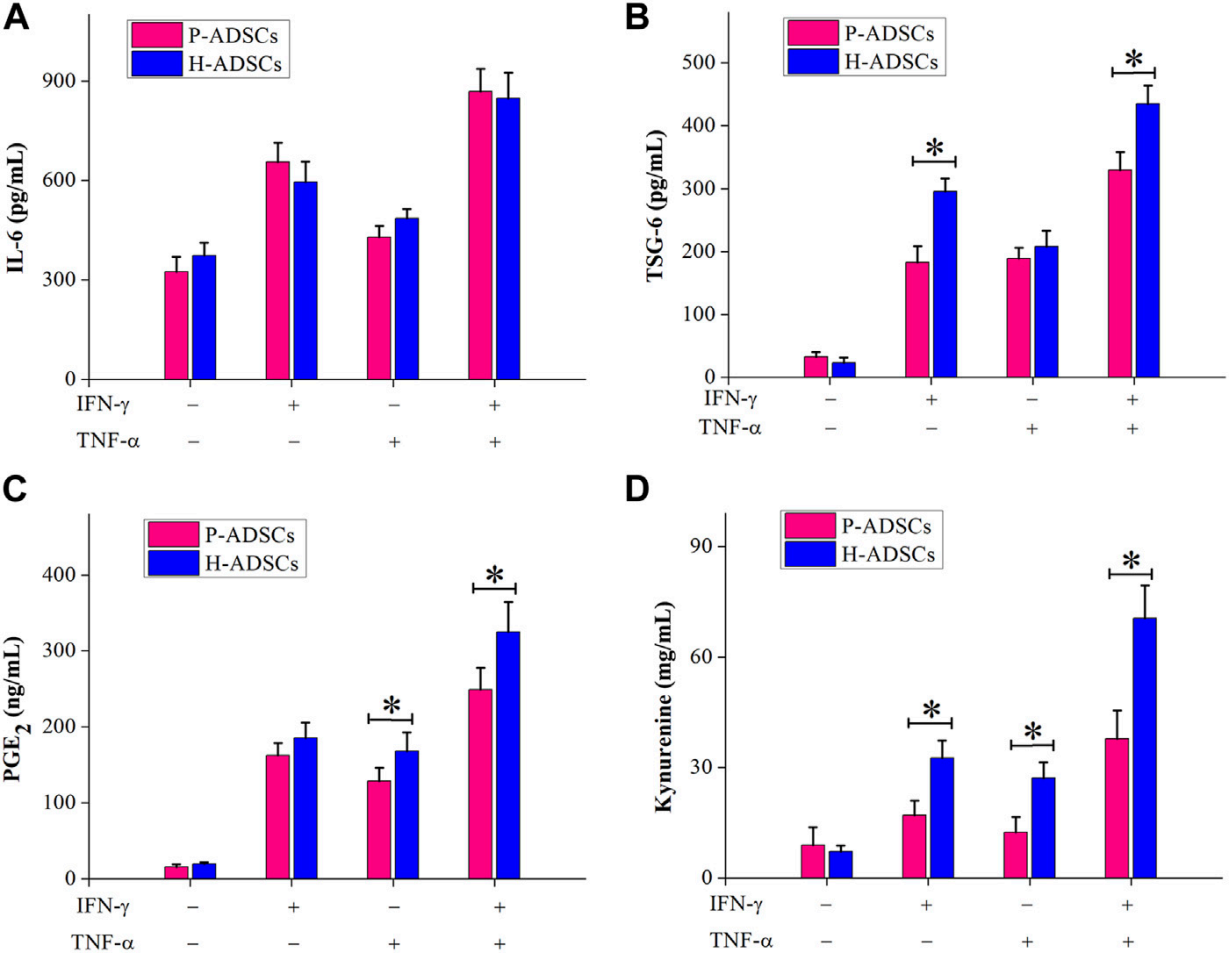
Immunosuppressive mediators involved in the immunosuppressive effect of P-ADSCs and H-ADSCs. P-ADSCs or H-ADSCs were primed with IFN-γ and TNF-α separately or combined for 48 h. Levels of the immunosuppressive factors IL-6 **(A)**, TSG-6 **(B)**, and PGE_2_ **(C)** determined by ELISA. **(D)** IDO activity expressed as the concentration of kynurenine (*n* = 7; *p < .05 determined by the Wilcoxon-Mann-Whitney nonparametric test).

Immunosuppression-mediating molecules were measured in P-ADSCs or H-ADSCs after priming IFN-γ and TNF-α separately or combined. With no treatment, there was a certain amount of IL-6 secretion by both P-ADSCs and H-ADSCs (Figure 5A), as well as secretion of TSG-6 (Figure 5B), PGE_2_ (Figure 5C), and IDO activity (Figure 5D). Upon treatment with IFN-γ or/and TNF-α, the secretion of IL-6, TSG-6, and PGE_2_, and IDO activity of P-ADSCs and H-ADSCs were increased. P-ADSCs primed with IFN-γ alone or with the combination of IFN-γ and TNF-α exhibited significantly lower TSG-6 secretion compared with H-ADSCs (both *p* < .05, Figure 5B). P-ADSCs primed with TNF-α alone or with the IFN-γ/TNF-α combination exhibited lower PGE_2_ secretion compared with H-ADSCs (both *p* < .05, Figure 5C). Moreover, P-ADSCs incubated with IFN-γ, TNF-α, or both exhibited lower IDO activity (all *p* < .05, Figure 5D), but IL-6 secretion was not significantly different between P-ADSCs and H-ADSCs (all *p* > .05, Figure 5A).

### Therapeutic Effects of P-ADSCs and H-ADSCs

**FIGURE 6:**
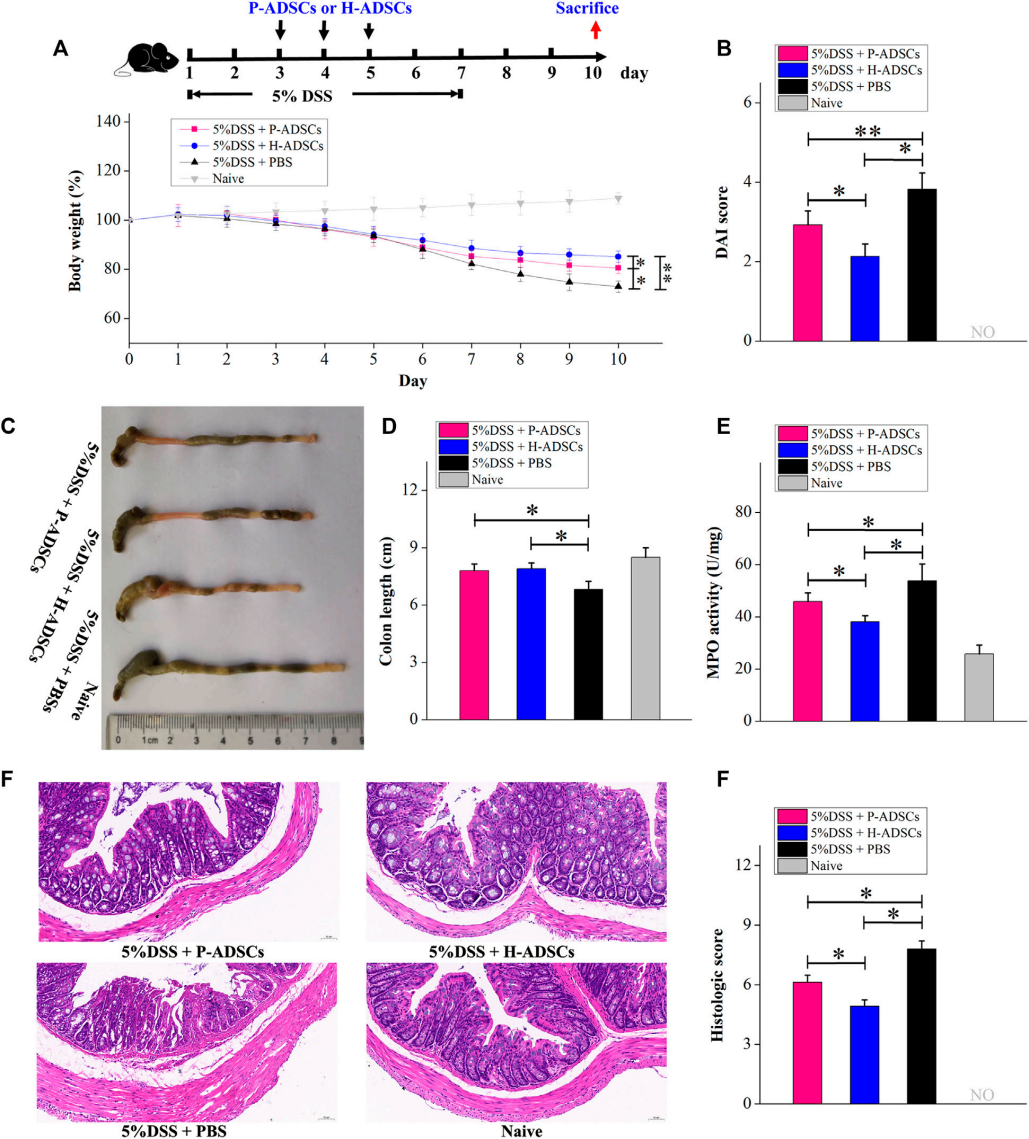
P-ADSCs exhibit weaker therapeutic effects in a mouse acute colitis model than H-ADSCs. **(A)**: Protocol for acute colitis induction and H-ADSCs or P-ADSCs injection. **(B)**: Changes in weight, measured daily and expressed as the percentage change from day 0. n ≥ 5 mice per group; *p < .05 and **p < .01 determined by the Kruskal–Wallis one-way analysis **(C)**: DAI scores on day 10. n ≥ 5 mice per group; *p < .05 and **p < 0.01 determined by the Kruskal–Wallis one-way analysis **(D)**: Colon lengths in different groups. Representative images are shown **(E)**: Colon lengths on day 10. n ≥ 5 mice per group; *p < .05 determined by the Kruskal–Wallis one-way analysis **(F)**: MPO activity in colons as a measure of neutrophil infiltration on day 10. n ≥ 5 mice per group; *p < .05 determined by the Kruskal–Wallis one-way analysis. **(G)**: Representative images of colon tissue sections using histological examination. Original magnification, ×200. **(H)**: Histological scores on day 10. n ≥ 5 mice per group; *p < .05 and **p < .01 determined by the Kruskal–Wallis one-way analysis.

To evaluate the therapeutic actions of P-ADSCs and H-ADSCs in acute colitis, we established an experimental mouse model of acute colitis after treatment with 5% DSS for 7 d and administered P-ADSCs or H-ADSCs three times between days 3 and 5 (Figure 6A). The weight of mice treated with P-ADSCs and H-ADSCs gradually increased between days 5–10 in comparison with the PBS control (P-ADSCs: *p* < .05; H-ADSCs: *p* < 0.01, Figure 6B), with recovery slower with P-ADSCs on day 10 than with H-ADSCs (*p* < .05, Figure 6B). Reduced DAI scores on day 10 were seen with both P-ADSCs and H-ADSCs (P-ADSCs: *p* < .05; H-ADSCs: *p* < .01, Figure 6C), although higher scores were seen in the P-ADSC group (*p* < .05, Figure 6C). At day 10, the colons of the P-ADSC and H-ADSC groups were significantly longer than in the control (both *p* < .05, Figures 6D,E), with little difference between the ADSC groups (*p* > .05, Figures 6D,E). Additionally, both the MPO activity (*p* < .05, Figure 6F) and histological scores (*p* < .05, Figures 6G,H) were markedly reduced in the H-ADSC-treated group relative to the P-ADSCs-treated group.

**FIGURE 7:**
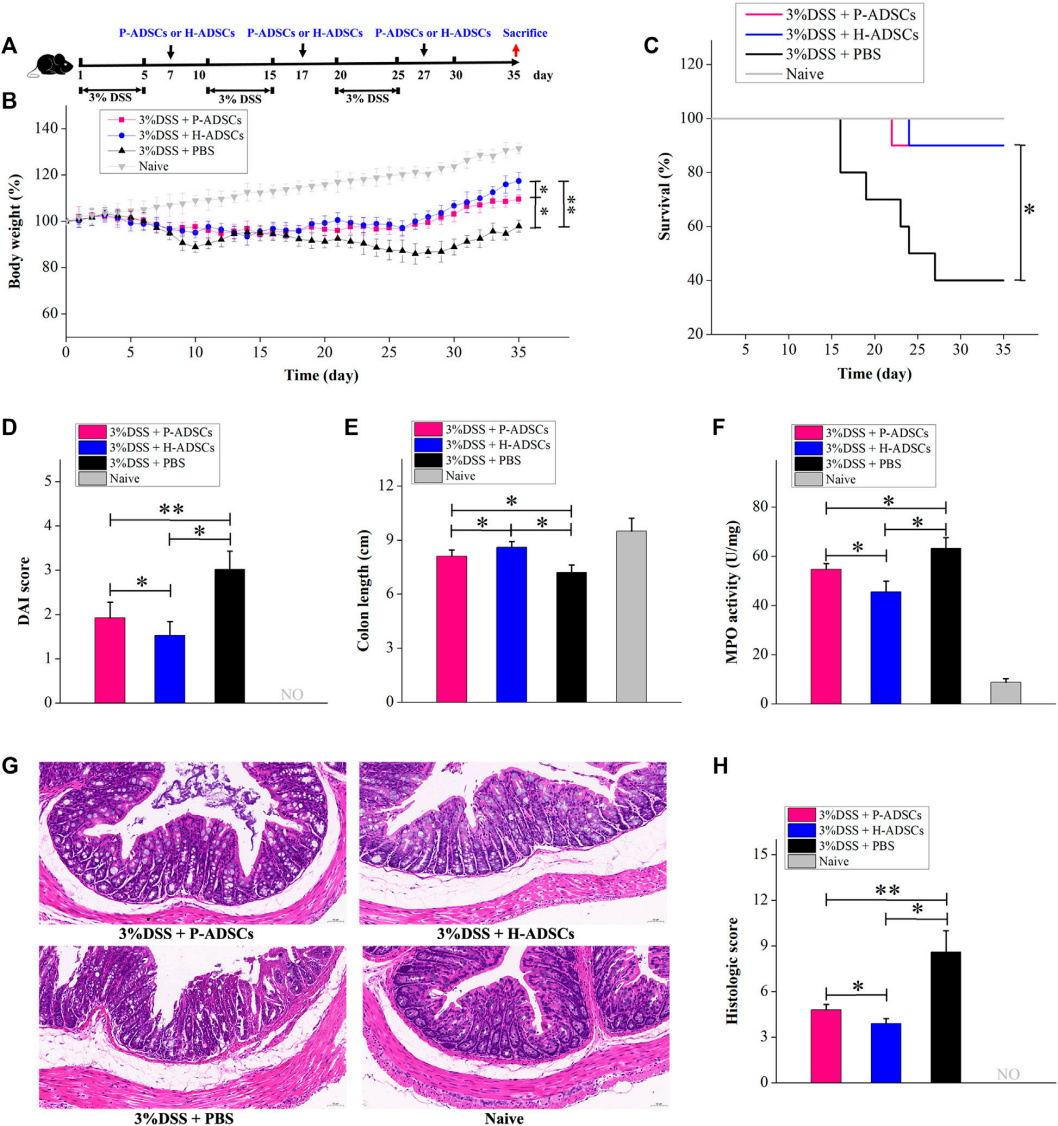
P-ADSCs exhibit weaker therapeutic effects in a mouse chronic colitis model than H-ADSCs **(A)**: Protocol for chronic colitis induction and H-ADSCs or P-ADSCs injection **(B)**: Changes in weight, measured daily and shown as percentage change from day 0. n ≥ 5 mice per group; *p < .05 and **p < .01 determined by the Kruskal–Wallis one-way analysis. **(C)**: Mortality was recorded. n ≥ 5 mice per group; *p < 0.05 determined by the Kruskal–Wallis one-way analysis. **(D)**: DAI scores were monitored on day 35. n ≥ 5 mice per group; *p < .05 and **p < .01 determined by the Kruskal–Wallis one-way analysis. **(E)**: Colon lengths on day 35. n ≥ 5 mice per group; *p < .05 determined by the Kruskal–Wallis one-way analysis. **(F)**: MPO activity in colons as a measure of neutrophil infiltration on day 35. n ≥ 5 mice per group; *p < .05 determined by the Kruskal–Wallis one-way analysis **(G)**: Representative images of colon tissue sections using histological examination. Original magnification, ×200 **(H)**: Histological scores on day 35. n ≥ 5 mice per group; *p < .05 and **p < .01 determined by the Kruskal–Wallis one-way analysis.

UC is a chronic disorder characterized by inflammation and long-term activation of immune cells, and it is possible that the immune reaction to ADSCs may differ in acute colitis produced by DSS. We further examined the therapeutic benefit of P-ADSCs and H-ADSCs in chronic colitis. Chronic colitis was induced by three cycles of 5-day administration of 3% DSS and a 5-day recovery between cycles and was followed by the administration of P-ADSCs or H-ADSCs three times on days 7, 17, and 27 (Figure 7A). The administration of H-ADSCs or P-ADSCs resulted in a significant improvement in clinical features such as weight loss (P-ADSCs: *p* < .05; H-ADSCs: *p* < .01, Figure 7B), mortality (both *p* < .05, Figure 7C), DAI (P-ADSCs: *p* < .05; H-ADSCs: *p* < .01, Figure 7D), colon shortening (both *p* < .05, Figure 7E), MPO activity (both *p* < .05, Figure 7F), and colon damage (P-ADSCs: *p* < .05; H-ADSCs: *p* < .01, Figures 7G,H) compared with PBS-treated mice. Notably, H-ADSC infusion was more effective in ameliorating disease severity than P-ADSC administration, seen in improved body weight (*p* < .05, Figure 7B) and DAI (*p* < .05, Figure 7D), as well as reduced colon shortening and MPO activity (both *p* < .05, Figures 7E,F), and reduced damage to the colon (*p* < 0.05, Figures 7G,H). However, there were no differences in survival between the P-ADSC and H-ADSC groups (*p* > .05, Figure 7C). Collectively, these results suggested that P-ADSCs had a certain therapeutic effect on acute and chronic UC, but the effect was weaker than that of H-ADSCs.

## Discussion

**FIGURE 8:**
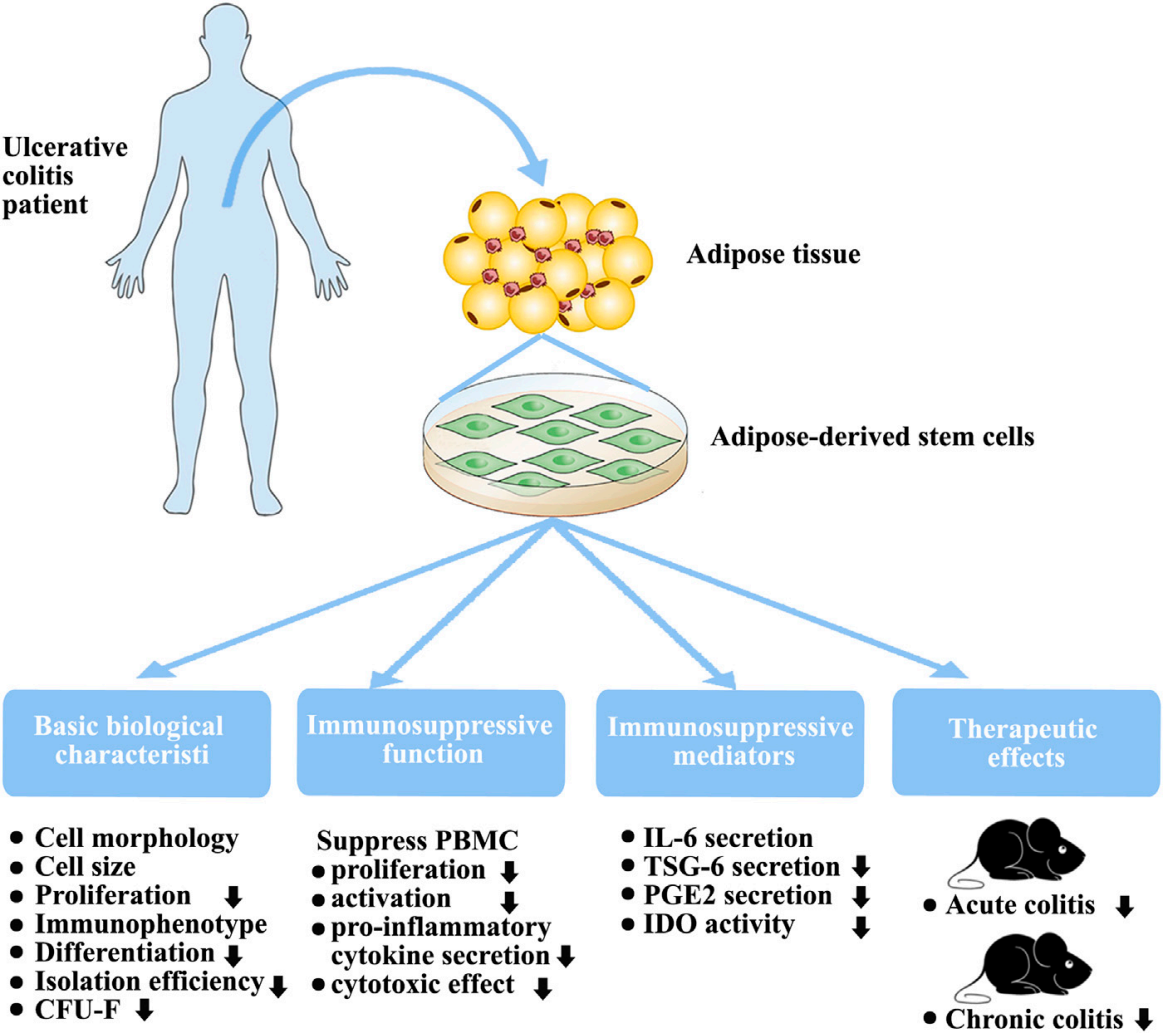
Biological characteristics, immunosuppressive function, and therapeutic effects of in vitro-expanded ADSCs from patients with UC.

When considering cellular therapy, autologous therapy is generally favored as it does not trigger immune rejection, despite the possibility of ADSC engraftment. However, there are concerns that ADSCs from patients with inflammatory and autoimmune conditions, including osteoarthritis (Skalska and Kontny, 2016), atherosclerosis (Kizilay Mancini et al., 2017), and rheumatic diseases (Kuca-Warnawin et al., 2019), may be defective in various aspects and may not be as effective as cells from healthy individuals. Recent evidence indicates that, together with the well-defined immune-related deficits in UC, AT may also be involved in the UC pathogenesis(Goncalves et al., 2015; Karaskova et al., 2021). UC leads to the formation of microenvironments that may adversely influence the functioning of various cell types, including myofibroblasts, stem cells, and epithelial cells within the crypts(Brown et al., 2014). In this study, the AT of all patients with UC could give rise to ADSCs. Furthermore, we found that culture-expanded H-ADSCs and P-ADSCs did not differ in their cell-surface markers, nor in cell morphology and size. However, our results revealed that P-ADSCs were present in lower proportions and had a lesser proliferative capacity and cloning ability than H-ADSCs (Figure 8). A previous study has demonstrated that pro-inflammatory cytokines (IFN-γ, TNF-α, and IL-1β) negatively modulate stem and progenitor cell proliferation after short- or long-term exposure(Kizil et al., 2015; Yang et al., 2018). This finding is consistent with our experimental results. However, only one group has observed that there are markedly more ADSCs in IBD patients than in those without IBD (patients with cancer)(Mizushima et al., 2018). In that study, the authors considered patients with cancer as the control group instead of healthy individuals, and patients in the IBD group were younger and had a lower BMI. Studies have reported that stem cells from young, thin donors have stronger proliferation ability(Serena et al., 2016; Myneni et al., 2018). These may also be the reasons for the inconsistencies with our experimental research results.

Differentiation capacity is a critical biological characteristic. Previous studies have demonstrated that some pro-inflammatory cytokines promote both osteogenesis and chondrogenesis after short-term treatment (Li et al., 2016; Yang et al., 2018) but a negative regulatory effect after long-term exposure(Liu et al., 2016). Here, we demonstrated that ADSCs from patients with UC of an average 34 months duration possessed lower osteogenic and chondrogenic differentiation capacities (Figure 8). These findings support those of other studies that showed that MSC differentiation capability is adversely affected by diabetes(Zhu et al., 2019).

Our main focus was on the immunomodulatory function of P-ADSCs. A major characteristic of MSCs is their immunomodulatory ability to suppress PBMC proliferation and activation, and reduce their pro-inflammatory cytokine secretions(Park et al., 2018; Lelek and Zuba-Surma, 2020). In our study, we demonstrated the impaired immunomodulatory function of P-ADSCs (Figure 8). Previous studies have observed that MSCs from newly diagnosed type 1 diabetes mellitus patients possess similar immunosuppressive characteristics to those from healthy individuals as they have not yet been exposed to the adverse metabolic and inflammatory aspects of diabetes(Yaochite et al., 2016). However, the therapeutic efficacy of MSC-derived exosomes was found to be reduced in type 1 diabetic rats(Zhu et al., 2019). Therefore, we speculated that these unfavorable environmental conditions induce changes in stem cells from diabetic patients initially to assist the immune response but with subsequent deleterious effects. A previous study demonstrated that MSCs from Crohn’s disease patients showed normal immunosuppression when used with high MSC:PBMC ratios and were more effective in inhibiting PBMCs proliferation when present in a low ratio (MSC:PBMC = 1:1000)(Bernardo et al., 2009). These results contradict our findings. Although the reason is not completely understood, three primary explanations are possible. First, although both UC and Crohn’s disease are classified as IBD, the immune responses of both in the body are not exactly the same. Crohn’s disease is mainly characterized by Th1 cell-mediated immune response, whereas Th1, Th2, and Th17 are involved in UC(De Francesco et al., 2016; Markovic et al., 2018). A study has shown that the cytokine environment affects the response by MSCs to the same stimulus, supporting the concept of complex immunoregulatory actions(Holan et al., 2016). Second, different culture conditions affect the immunosuppressive functions of MSCs. In our study, we used hPL medium instead of traditional fetal bovine serum as a medium. Studies have reported that hPL influences the repressive effects of MSCs on T cells and NK cells(Abdelrazik et al., 2011). Lastly, different tissue sources affect the MSC immunosuppressive action. In our laboratory, we observed that, when cultured with hPL, BMMSCs and ADSCs exhibited different biological characteristics and different immunosuppressive functions(Li et al., 2015). The therapeutic action of MSCs in inflammation is a consequence of their reduced immunogenicity and immunomodulatory abilities(Jiang and Xu, 2019; Hosseini-Asl et al., 2020). MSCs are primarily used in IBD because of their capacity to suppress immune activity. Furthermore, our findings suggested that ADSCs derived from individuals with UC have limited potential in a DSS-induced animal model of acute or chronic colitis (Figure 8), which we mainly attribute to their impaired immunosuppressive capabilities. Our experimental results are contrary to those of some studies evaluating the effect of short-term pretreatment of MSCs with inflammatory factors *in vitro*(Fuenzalida et al., 2016; Cheng et al., 2017; Qiu et al., 2017). Although the reason why the function of ADSCs is decreased in UC is not fully known, there are two probable explanations. The first may be related to the complex inflammatory state *in vivo*. The other explanation relates to the long-term actions of pro-inflammatory factors *in vivo*. Studies show that specific cytokines vary in their effects on MSC immunoregulatory properties, and that long-term exposure may adversely influence MSC immunomodulation(Holan et al., 2016). We speculated that these adverse environmental conditions may affect ADSCs from UC patients initially to assist the immune response but with long-term deleterious effects(Yang et al., 2018).

MSCs regulate immune responses by releasing various modulatory factors including PGE_2_, IL-6, TSG-6, and IDO(Shi et al., 2018; Jiang and Xu, 2019). Our results showed marked differences in the secretion of these factors between P-ADSCs and H-ADSCs (Figure 8). P-ADSCs, both treated and untreated, exhibited similar levels of IL-6 secretion, but less of TSG-6, PGE_2_, and IDO. Our findings agree with previous reports that IL-6 and IDO in ADSCs from patients with rheumatic diseases (Kuca-Warnawin et al., 2019); however, the results of TSG-6 and PGE_2_ are inconsistent and the results of TSG-6 are contradictory(Kuca-Warnawin et al., 2019). A possible reason for this discrepancy is that UC and rheumatoid arthritis may be similar inflammatory disorders but may elicit different immune responses. These various ADSC factors may stimulate pathways to regulate the actions of MSCs and may be responsible for the therapeutic efficacy in animal colitis models. Frequently, combinations of these factors are necessary to achieve immunosuppression. Although the reason why UC impairs ADSC release is not completely understood, it is possible that P-ADSCs may become tolerant of cytokines under long-term disease conditions. Nevertheless, it is notable that impairment of these mediators by ADSCs may be responsible for the poor regulation of systemic inflammation in UC.

Our study has some limitations. First, all fat samples in this study were obtained from healthy controls matched by age and gender, which are comparable. Only a small number of patient specimens were obtained for this study. The results may have sampling errors, which makes this study have a limited reference value. Second, the detailed mechanisms of the impaired immunomodulatory function of ADSCs in UC have not been elucidated. Further investigation is necessary to compare the effect of ADSCs in large sample sizes and to elucidate their detailed mechanisms of action. In addition, the impaired immunomodulatory function of ADSCs from UC patients still does not rule out completely the possibility of an impact of the systemic immunosuppressive treatment. One would expect a decrease in the immune response because of the immunosuppressant properties of drugs used in patients with UC; However, clinically relevant doses of immunosuppressive drugs including glucocorticoids interfere with ADSCs functions, but enhance or at least do not restrain their immunosuppressive properties (Girdlestone et al., 2015; Javorkova et al., 2018).

Here, we have shown, for the first time, that the immunomodulatory function of ADSCs is impaired in UC. Further strategies to improve immunosuppressive properties are required before using ADSCs derived from patients with UC. Patients with UC are usually in poor health conditions and unable to tolerate the harvest of a large amount of AT. Thus, in such patients, autologous ADSC transplantation may be therapeutically ineffective.

## Data Availability

The original contributions presented in the study are included in the article/Supplementary Material, further inquiries can be directed to the corresponding authors.
