# Supplementary material for: Adipose-Derived Stem Cells From Patients With Ulcerative Colitis Exhibit Impaired Immunosuppressive Function
Source: Front Cell Dev Biol. 2022 Feb 18;10:822772. doi: 10.3389/fcell.2022.822772 (PMC8894714; doi:10.3389/fcell.2022.822772)
Supplement: Supplementary file 1 [file DataSheet1.docx]

Supplementary Figure 1. Gating strategy for the experiment with ADSCs phenotype.


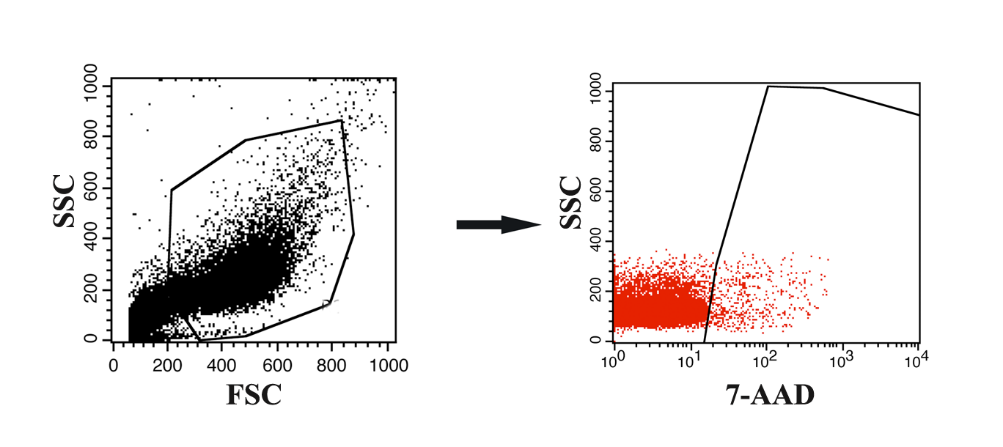


Supplementary Figure 2. Gating strategy for the experiment with PBMCs activation status.


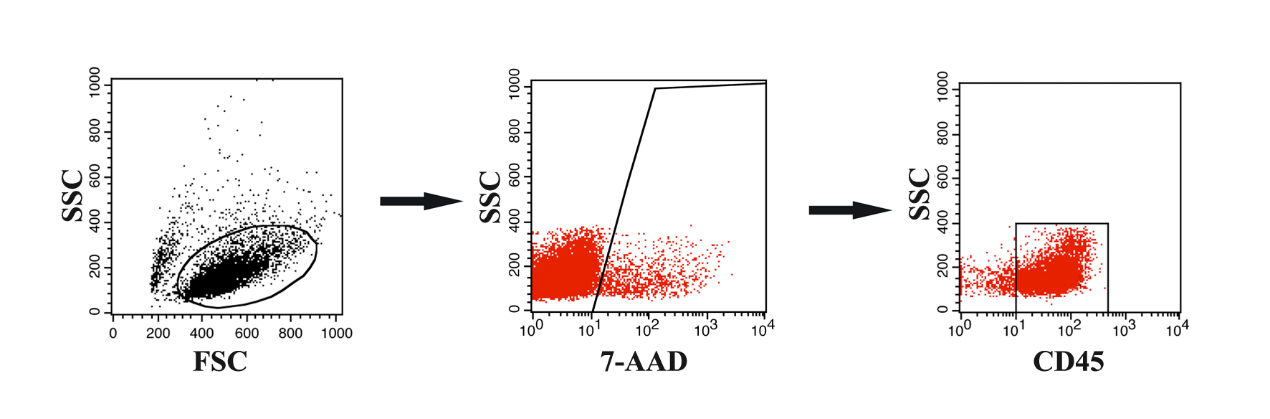


Supplementary Table 1. The clinical characteristics of the patients

| Patient  number | Age (year) | Sex | Body mass index (kg/m^2^) | Duration  (months) | Disease  degree | Therapy |
| --- | --- | --- | --- | --- | --- | --- |
| 1 | 43 | Male | 23.91 | 36 | Severe | Mesalazine |
| 2 | 39 | Female | 24.09 | 21 | Mild | Mesalazine |
| 3 | 47 | Male | 18.67 | 40 | Severe | Mesalazine |
| 4 | 52 | Female | 22.31 | 36 | Moderate | Mesalazine |
| 5 | 63 | Female | 21.82 | 30 | Moderate | Mesalazine |
| 6 | 40 | Female | 19.16 | 42 | Severe | Methylprednisolone |
| 7 | 46 | Male | 20.62 | 28 | Moderate | Mesalazine |

Supplementary Table 2. Flow cytometry antibodies from BD bioscience

| Antibody | Clone | Catalog number |
| --- | --- | --- |
| PE Mouse Anti-Human CD73 | AD2 | 550257 |
| FITC Mouse Anti-Human CD90 | 5E10 | 555595 |
| PerCP-Cy™5.5 Mouse anti-Human CD105 | 266 | 560819 |
| PerCP-Cy™5.5 Mouse Anti-Human CD14 | M5E2 | 550787 |
| FITC Mouse Anti-Human CD19 | HIB19 | 560994 |
| PE Mouse Anti-Human CD34 | 581 | 555822 |
| FITC Mouse Anti-Human CD45 | HI30 | 561865 |
| APC Mouse Anti-Human HLA-DR | G46-6 | 560744 |
| PE Mouse Anti-Human CD25 | M-A251 | 560989 |
| FITC Mouse Anti-Human CD69 | FN50 | 555530 |

Supplementary Table 3. Primer sequences for Real Time PCR analysis and their respective product sizes.

| Gene | Primer sequence (5' to 3') | Product size (bp) | NCBI Reference Sequence |
| --- | --- | --- | --- |
| RUNX-2 | GGAGTGGACGAGGCAAGAGTTT | 133 | NM_001015051.4 |
|  | AGCTTCTGTCTGTGCCTTCTGG |  |  |
| AKP | GGGAACGAGGTCACCTCCAT | 67 | NM_001127501.4 |
|  | TGGTCACAATGCCCACAGAT |  |  |
| SOX-9 | GTACCCGCACTTGCACAAC | 139 | NM_000346.4 |
|  | GTAATCCGGGTGGTCCTTCT |  |  |
| Collagen II | GGCAATAGCAGGTTCACGTACA | 79 | NM_033150.3 |
|  | CGATAACAGTCTTGCCCCACTT |  |  |
| PPARγ | GGCTTCATGACAAGGGAGTTTC | 74 | NM_001354666.3 |
|  | AACTCAAACTTGGGCTCCATAAAG |  |  |
| LPL | GAGGTACTTTTCAGCCAGGATGTAAC | 82 | NM_000237.3 |
|  | AGCTGGTCCACATCTCCAAGTC |  |  |
| β-actin | TGACGTGGACATCCGCAAAG | 205 | NM_001101.5 |
|  | CTGGAAGGTGGACAGCGAGG |  |  |

Supplementary Table 4. The disease activity index scoring, based on the clinical sign of colitis

| Score | Bleeding | Stool consistency | Body weight changes |
| --- | --- | --- | --- |
| 0 | Negative | Formed | With no change/increase |
| 1 | Positive occult blood test | Soft | 1%–5% decrease |
| 2 | Visible bleeding | Loose | 6%–10% decrease |
| 3 | Severe bleeding | Watery | 11%–20% decrease |
| 4 | - | - | More than 20% decrease |

Supplementary Table 5. Colonic Histological Scoring System

| Histological feature | Score | Description |
| --- | --- | --- |
| Epithelial damage | 0 | None |
|  | 1 | 0%–5% loss of epithelium |
|  | 2 | 5%–10% loss of epithelium |
|  | 3 | Over 10% loss of epithelium |
| Loss of crypts | 0 | None |
|  | 1 | 0%–10% loss of crypts |
|  | 2 | 10%–20% loss of crypts |
|  | 3 | Over 20% loss of crypts |
| Infiltration of inflammatory cells | 0 | None |
|  | 1 | Mild (10%) |
|  | 2 | Moderate (25%) |
|  | 3 | Severe (40%) |
